# A Five-Decade Text Mining Analysis of Cochlear Implant Research: Where We Started and Where We Are Heading

**DOI:** 10.3390/medicina59111891

**Published:** 2023-10-24

**Authors:** Idit Tessler, Nir A. Gecel, Benjamin S. Glicksberg, Shaked Shivatzki, Yisgav Shapira, Eyal Zimlichman, Eran E. Alon, Eyal Klang, Amit Wolfovitz

**Affiliations:** 1 Department of Otolaryngology and Head and Neck Surgery, Sheba Medical Center, Ramat Gan 52621, Israelshaked.shivatzki@gmail.com (S.S.); yisgav.shapira@sheba.health.gov.il (Y.S.); eran.alon@sheba.health.gov.il (E.E.A.); amit.wolfovitz@gmail.com (A.W.); 2 ARC Innovation Center, Sheba Medical Center, Ramat Gan 52621, Israel; eyal.zimlichman@sheba.health.gov.il (E.Z.); eyalkla@hotmail.com (E.K.); 3 Hasso Plattner Institute for Digital Health, Icahn School of Medicine at Mount Sinai, New York, NY 10029, USA

**Keywords:** machine learning, artificial intelligence, cochlear implant, indication, hearing preservation, pediatrics, robotic, hearing loss

## Abstract

*Background and Objectives*: Since its invention in the 1970s, the cochlear implant (CI) has been substantially developed. We aimed to assess the trends in the published literature to characterize CI. *Materials and Methods*: We queried PubMed for all CI-related entries published during 1970–2022. The following data were extracted: year of publication, publishing journal, title, keywords, and abstract text. Search terms belonged to the patient’s age group, etiology for hearing loss, indications for CI, and surgical methodological advancement. Annual trends of publications were plotted. The slopes of publication trends were calculated by fitting regression lines to the yearly number of publications. *Results*: Overall, 19,428 CIs articles were identified. Pediatric-related CI was the most dominant sub-population among the age groups, with the highest rate and slope during the years (slope 5.2 ± 0.3, *p* < 0.001), while elderly-related CIs had significantly fewer publications. Entries concerning hearing preservation showed the sharpest rise among the methods, from no entries in 1980 to 46 entries in 2021 (slope 1.7 ± 0.2, *p* < 0.001). Entries concerning robotic surgery emerged in 2000, with a sharp increase in recent years (slope 0.5 ± 0.1, *p* < 0.001). Drug-eluting electrodes and CI under local-anesthesia have been reported only in the past five years, with a gradual rise. *Conclusions*: Publications regarding CI among pediatrics outnumbered all other indications, supporting the rising, pivotal role of CI in the rehabilitation of children with sensorineural hearing loss. Hearing-preservation publications have recently rapidly risen, identified as the primary trend of the current era, followed by a sharp rise of robotic surgery that is evolving and could define the next revolution.

## 1. Introduction

Hearing impairment is not just a physical condition; it is a pervasive issue that affects social, emotional, psychological, and physical wellbeing. As the most common sensory deficit, it has far-reaching consequences and impacts over 466 million people globally [1]. It can lead to social isolation, difficulty in communication, reduced job performance, and, in severe cases, even cognitive decline.

Since its groundbreaking introduction in 1971, cochlear implants (CIs) have revolutionized the field of otolaryngology and audiology by offering a functional replacement for damaged sensory hair cells. They have become the preferred treatment for patients with severe to profound sensorineural hearing loss (SNHL) [2]. Unlike hearing aids, which amplify sound, CIs directly stimulate the auditory nerve, with the ability to discriminate different sound frequencies. Today, CIs are recognized as top-tier neurobionic prostheses, being able to replicate complex human sensory functions [3].

Over the past few decades, CI technology underwent significant advancements to meet the needs of patients. While early versions of CIs were relatively simple, offering limited sound perception, modern CIs are sophisticated devices with multiple channels, allowing for better sound quality and tonotopic mapping. Surgical techniques have also evolved, with a focus on minimizing invasiveness and preserving residual hearing. New objectives like reducing cochlear trauma and inner ear inflammation are now evolving with the development of innovative biomaterials and nanomaterials [4].

In the past years, there has been ongoing widening of the indications for CI usage, both in terms of target populations and indications. No longer restricted to severe cases or specific age groups, CIs are now being explored for patients with single-sided deafness, asymmetric hearing loss, and indications other than SNHL, such as tinnitus. This signals a paradigm shift in how we approach auditory rehabilitation.

In the era of big data, text mining has emerged as an analytic tool for researchers. It employs different techniques to extract vital data and insights such as natural language processing, machine learning, and statistical analysis to extract valuable information and insights from large volumes of unstructured text. Unlike traditional literature reviews, which often rely on manual scrutiny of a limited number of papers, text mining enables the automated analysis of thousands of articles, thereby allowing for analysis of thousands of articles from a “bird’s-eye” perspective [5,6]. This approach can identify hidden patterns, trends, and connections in large datasets, providing insights that may not be immediately apparent. Text mining has a broad range of applications, from market research to social sciences. This technique is increasingly being used in medical research to analyze the large literature datasets [6,7,8,9,10,11]. In the context of our study, text mining was instrumental in parsing and analyzing the vast corpus of CI-related publications. Guided via a curated taxonomy and a set of pre-defined topics and search terms, we aimed at categorizing publications spanning five decades in the field of CIs. This methodical approach enabled us to delineate distinct trends and shifts in the focus of CI research over time. By systematically organizing this extensive body of literature, we are able to gain insights into evolving research interests, technological advancements, and changing demographics of patients benefiting from CIs. This categorization served as a robust framework for exploring the longitudinal trends, thereby shedding light on the past, present, and potentially, the future trajectory of CI research.

The evolution of CI technology and its application has markedly influenced the management of hearing loss. It has transformed treatment options and approaches for both congenital and acquired deafness. However, the pace at which these advancements are occurring is rapid, and keeping track of the latest trends is challenging. Text mining offers a solution to this challenge by enabling us to identify research trends based on publication trends. In this study, we utilize text mining to examine the literature on CIs over the past five decades, aiming to reveal the evolving focus of this crucial area of medical science.

### 1.1. Cochlear Implant

#### 1.1.1. Historical Evolution: A Journey from Single- to Multi-Channel Devices

The history of CIs is an interesting narrative of scientific innovation [12]. The first CI was implanted by William House and John Doyle of Los Angeles, California, in 1961, followed by Blair Simmons and Robert White of Stanford University, Stanford, California, who inserted a six-channel electrode into the modiolus in 1964 [13,14].

But, it was the innovative work of two separate teams led by Graeme Clark in Australia and Ingeborg Hochmair in Austria that changed the landscape of auditory prostheses [15,16]. These teams independently developed multi-channel implants in the late 1970s, thus providing nuanced auditory perception superior to their single-channel predecessors [16,17,18,19]. CI progress continued since then. From body-worn processors in 1994 to behind-the-ear versions, the hardware underwent significant advancement [16,20]. As of 2019, more than 200,000 people in the United States have benefited from this transformative technology [21]. This number is consistently rising, which reflects the global acceptance and utilization of CIs [21].

#### 1.1.2. Technological Advancements: A Symphony of Microelectronics and Biotechnology

Modern CIs are a testament of interdisciplinary collaboration, integrating insights from audiology, neuroscience, electrical engineering, and materials science [22,23]. The external sound processor has evolved from a simple microphone to sophisticated systems that employ digital signal processor (DSP) chips [24,25]. These chips execute complex algorithms that filter and prioritize audible speech, providing a more refined auditory experience for the user [17,25].

Internally, the electrode array inserted into the cochlea has also been improved significantly [26]. While early designs were relatively crude, advancements in biocompatible materials and nanotechnology have resulted in units that minimize internal scarring and inflammation [17,27]. These innovations aim to preserve residual hearing and provide a more comfortable experience for recipients [17,27]. The development of totally implantable cochlear implants (TICIs) is another breakthrough on the horizon. TICIs aim to integrate all external components into the internal implant. This will make the device less visible and more resilient to external environmental factors [28,29].

The fundamental workings of cochlear implants are important to understand in order to fully appreciate the technology’s transformative impact on the field of auditory sciences. Unlike conventional hearing aids, which amplify external sounds, a CI functions by bypassing the damaged parts of the auditory system to directly stimulate the auditory nerve. The CI system comprises two main components: an external sound processor that captures and processes sound, and an internal implant with an electrode array surgically inserted into the cochlea. The sound processor captures ambient sounds and converts them into digital signals. These signals are then transmitted to the internal implant, which sends electrical impulses via the electrode array to different regions of the auditory nerve. The auditory nerve transmits these signals to the brain, where they are interpreted as sound. This technology effectively restores a form of hearing to individuals with severe to profound sensorineural hearing loss.

#### 1.1.3. Surgical Procedures and Safety Metrics: An Evolution towards Minimized Risks

The surgical implantation process for cochlear implants, typically performed under general anesthesia, has also experienced significant progress [24,27]. While the procedure involves a mastoidectomy and a facial recess approach, evolving techniques focus on reducing cochlear trauma and inflammation [27,30]. Surgical protocols now include the use of imaging technologies to plan the implantation better, thereby reducing the risk of complications which include facial nerve damage and cochlear injury [27,30].

Over the years, complication rates have drastically reduced [30,31]. While in the early 1990s, the complication rate was over 35%, this rate has plummeted to less than 10% due to enhanced surgical techniques and better post-operative care. The dramatic reduction in complications shows the strides made in making the procedure safer and more effective for a broad range of patients [30,32]. Local anesthesia instead of the traditional general anesthesia in another developing option for increasing safety and expanding the target population, especially among the elderly.

#### 1.1.4. Patient Outcomes: Individual Variances and Influencing Factors

The outcomes for adult and pediatric cochlear implant patients are favorable [33]. However, while cochlear implants have obvious positive outcomes, we should recognize the range of individual experiences post-implantation [34,35]. Factors such as the patient’s age at implantation, duration of deafness, and overall health can significantly influence the outcomes [34,35]. Moreover, the level of parental engagement in the case of children, educational background, and even the specific positioning of the implant within the cochlea may also play a role [36].

Despite these variations, a majority of patients experience significant improvements in auditory perception, speech recognition, and overall quality of life [22,34]. These improvements are supported by a large base of scientific studies and meta-analyses, reinforcing the efficacy of CIs as a life-changing intervention [22].

#### 1.1.5. Expanding Demographics: From Severe Loss to Unilateral Deficiencies

The initial target population for cochlear implants was patients with severe to profound bilateral hearing loss [20,22]. However, ongoing research is looking at the benefits of cochlear implants for a broader range of auditory disorders. These include conditions such as single-sided deafness and complex cases of auditory neuropathy spectrum disorder [19,37]. As our understanding deepens, it is likely that CIs will become a viable solution for an increasingly diverse set of hearing impairments, thereby expanding their impact and utility [19,37].

#### 1.1.6. The Economic and Societal Impact: A Worthwhile Investment

The value of CIs extends beyond the individual to the broader society. In the United States, insurance coverage for CIs has expanded, reflecting their acceptance as a valuable and cost-effective medical intervention [21]. Globally, the World Health Organization (WHO) has recognized CIs as a cost-effective treatment for hearing loss, with marked returns on investment ranging from 1.46 to 4.09 dollars for every dollar spent. These economic returns translate into substantial public health advantages, particularly where untreated hearing loss costs globally nearly a trillion dollars annually.

## 2. Materials and Methods

The methodology employed in this study was designed to provide an understanding of CI literature trends on the pre-defined aspects. We initiated our study by retrieving all articles from PubMed and classifying the entries by key terms. We then applied a set of inclusion criteria and leveraged a taxonomy developed by expert otolaryngologists to categorize articles into four high-level categories: patient’s age group, hearing-loss etiology, indication for CI, and surgical methods. This taxonomy allowed us to perform a structured analysis of the thematic content in the collected articles. In addition, we classified journals to assess the interdisciplinary reach of CI research. Finally, we utilized statistical tools to analyze and visualize temporal trends in the publication. The following sections detail each of these methodological steps.

### 2.1. Data Collection

To comprehensively examine publications related to CIs, we systematically collected data from the PubMed database, which contains over 30 million citations for biomedical literature. PubMed is maintained by the National Center for Biotechnology Information (NCBI) at the United States National Library of Medicine (NLM) and allows free public access to MEDLINE records as well as publisher-supplied citations.

On 11 July 2022, we executed a detailed search query using PubMed’s public application programming interface (API). The API provided access to retrieve key metadata fields for each article indexed in PubMed, including the unique PubMed ID, publication year, journal name, article title, keywords, and abstract text. These metadata elements enabled both high-level bibliometric analysis as well as the granular assessment of abstract contents. Custom Python scripts were developed to programmatically interact with PubMed’s API to collect these data fields at scale for all relevant CI articles.

### 2.2. Inclusion Criteria and Search Strategy

Our search criteria were designed to be highly inclusive, identifying all PubMed articles pertaining to CIs. Specifically, we searched article titles, abstracts, and keywords for the terms “cochlear implant” or “cochlear implantation”. We included matches dated from 1 January 1970 to 11 July 2022. Using this approach, we obtained data capturing over 50 years of CI literature.

### 2.3. Article Classification and Keyword Mapping

To enable structured analysis of abstract contents, two expert otolaryngologists reviewed the CI literature to identify key terms frequently used in this domain. Through consensus, they compiled a comprehensive list of relevant keywords and organized these into a taxonomy encompassing four high-level categories: *patient’s age group, hearing-loss etiology, indication for CI, and surgical methods*.

In constructing the categories, we aimed to cover essential clinical aspects of CI research that would offer a focused view of the field’s advancements over five decades. To this end, we carefully selected four categories that are both clinically and methodology relevant:

**Patient’s Age Group:** Age is an important factor in cochlear implant outcomes and healthcare decision-making. The age groups eligible for CI have substantially developed over the years. By examining the literature through this lens, we can better understand how age-specific treatments have evolved and how the demographics of CI recipients have changed over time.

**Hearing-Loss Etiologies**: Understanding the root causes of hearing loss and the etiology for implantation is crucial for effective CI treatment. This category allows us to explore the expanding research focus on various etiologies over the years. It also offers insights into possible connections between advancements in diagnostics and genetics and the impact on CI applications.

**Indications for CI**: This category explores the clinical reasons for undergoing CI. Over the years, the indications for CI have expanded, thanks in part to technological advancements and a deeper understanding of auditory pathology. 

**Surgical Methods**: Surgical techniques have a direct impact on patient outcomes and the effectiveness of the implant. This category enables us to trace the technological and procedural innovations that have been published and identify potential rising trends.

Each of these categories was selected not just for its individual relevance but also for its ability to provide a multi-dimensional view of the CI field when analyzed altogether. This categorical framework serves as the backbone of our text-mining analysis, allowing us to present a focused yet inclusive review of CI research spanning five decades.

This expert-guided keyword list provided a method to classify the focus of each article based on the title and abstract text. Two otolaryngologists mapped the curated keywords to one of the four pre-defined categories. By annotating each abstract with applicable keyword labels, we could categorize the thematic contents based on the presence of the patient’s age group, hearing-loss etiology, indication for CI, and surgical methods keywords. The full list of keywords organized into the four categories is presented in Table 1.

### 2.4. Journal Classification

To better understand the interdisciplinary reach of CI research, we also classified the journals associated with each article based on their primary field of study. Using Scimago Journal Rankings as a reference (https://www.scimagojr.com/ (accessed on 18 August 2022)), we identified journals specifically focused on Otorhinolaryngology or Speech and Hearing, categorizing these as ENT-related journals for the purposes of this study. This journal classification allowed us to quantify and compare the overall publication trend within and beyond the core ENT literature.

### 2.5. Statistical Analysis

All data processing and statistical analyses for this study were performed using Python (Python Software Foundation, Version 3.6.5).

We utilized Python’s Pandas library for computing summary statistics. Categorical variables are summarized using counts and percentages. For non-categorical variables, such as publication counts over time, we present descriptive statistics such as means and variability measures where appropriate.

Temporal publication trends are visualized graphically using Python’s matplotlib and Seaborn libraries. To quantify the precise rate of publication growth over time, we employ linear regression, fitting a straight line model with the year of publication (X) predicting the publication count (Y). We assess the statistical significance of the temporal increase by computing *p*-values for each fitted regression slope, with *p* < 0.05 indicating significant growth.

## 3. Results

*Publication Yield and Timeline:* Our search within the PubMed database yielded a total of 19,428 CI-related publications spanning from 1970 to 2022.

**Figure 1:**
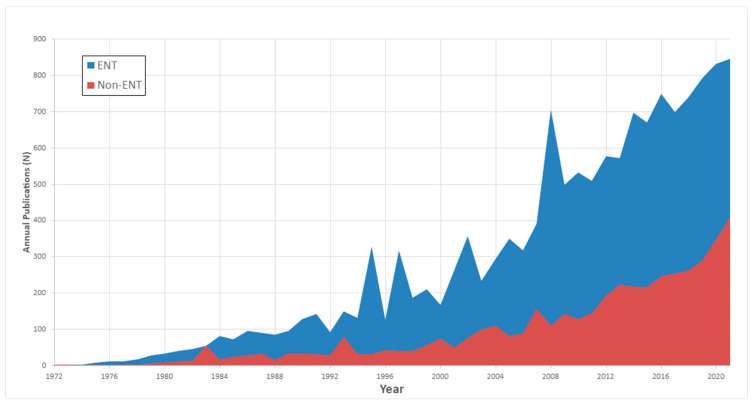
Distribution of CI’s publications according to the type of journal: Otolaryngology vs. not- Otolaryngology.

*Journal Distribution:* The distribution of these publications across ENT-specific and non-ENT journals is depicted in Figure 1. An examination of the temporal distribution highlighted a substantial growth in the diversity of publishing journals. Specifically, there was a notable increase in the number of journals publishing on this topic, with the count quadrupling from 335 journals during the first three decades (1970–2000) to 1237 journals in the last two decade (2001–2022) timeframe.

### 3.1. Publication Trends

**Figure 2:**
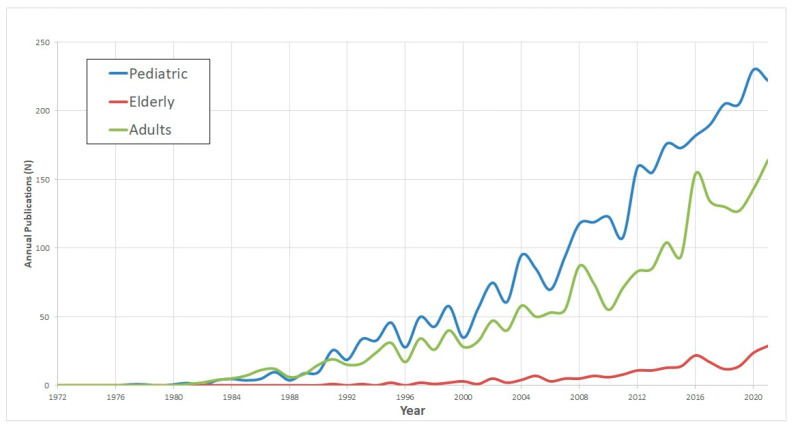
Cochlear implant 5-decade demographic publications trend.

**Figure 3:**
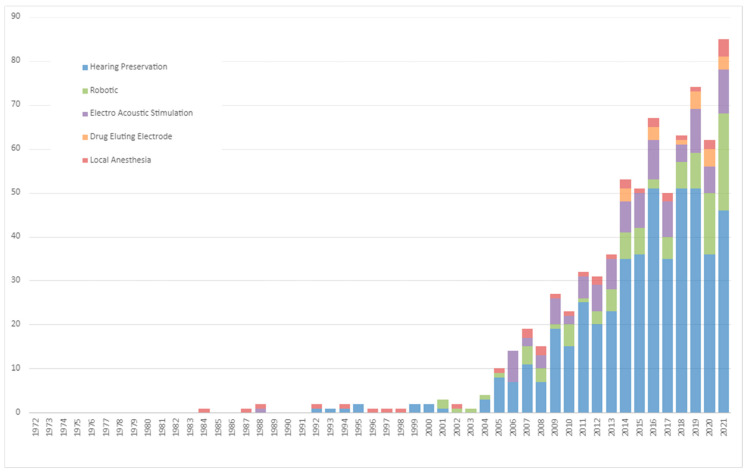
Five Decades of Surgical Methods Trends in Cochlear Implantation.

Publications trends in CI research related to the patient’s age group, hearing-loss etiology, indication for CI, and surgical methods are delineated in Figure 2 and Figure 3 and Table 2.

*Pediatric Research:* Among these, pediatric CI research stands out as the most prolific area, accounting for 3451 publications, which translates to 17.8% of the entire dataset. This domain showed the sharpest growth trajectory with a rate of 5.2 ± 0.3 (*p* < 0.001).

*Research on the Elderly:* In contrast, studies focusing on CI in elderly populations emerged more recently. This topic began gaining research traction from 2005 onwards. Despite its later onset, there has been a discernible uptick in publications, albeit at a more conservative growth rate of 0.7 ± 0.1 (*p* < 0.001).

### 3.2. Research Topics

*Etiologic Considerations:* The last ten years have evidenced an uptick in research articles focused on specific etiologies with tinnitus leading. Publications centered on tinnitus raised at a rate of 0.8 ± 0.1 (*p* < 0.001) during the study period. Genetic hearing loss was not far behind, growing at 0.7 ± 0.1, followed closely by infections and auditory neuropathy spectrum disorder, both rising at a pace of 0.6 ± 0.1. (Table 2)

*Indications for CI Trends:* In examining the indications for Cis, asymmetric hearing loss (AHL) research clearly stands out. Publications in this domain expanded at a rate of 0.5 ± 0.1 (*p* = 0.002). In contrast, research on single-sided deafness (SSD) experienced a peak around 2015 with a growth of 1.0 ± 0.6 (*p* = 0.333), only to taper off in following years.

*Surgical Methodological Advancements:* When considering the CI surgical methodologies depicted in Figure 3, hearing preservation has been at the forefront, showing the steepest growth at 1.7 ± 0.2 (*p* < 0.001). Robotic-assisted surgery, a relatively modern approach in the CI surgical spectrum, also demonstrated a rapid rise, growing at a rate of 0.5 ± 0.1 (*p* < 0.001). Two budding trends, drug-delivering electrodes and the use of local anesthesia, have started making their presence felt, albeit currently registering only modest growth trajectories.

## 4. Discussion

Cochlear implantation technology has undergone a rapid evolution since its inception. This solidifies its role as the primary rehabilitative treatment for severe to profound sensorineural hearing loss [1,2,3]. Initially, CI was a groundbreaking concept; today, it has matured into a sophisticated medical intervention with far-reaching implications. One of the important advancements in this field was the transition from a single-channel implant to a multichannel one. This technological leap has transcended the boundaries of mere auditory perception to significantly improve patients’ social interactions, academic performance, and overall quality of life [38,39].

In the current study, we sought to provide a targeted overview of the developments in specific aspects in CI research over the past five decades. We explored pre-defined facets of CI research including the patient’s age group, hearing-loss etiology, indication for CI, and surgical methods.

The demographic trends revealed in our study are consistent with the developments in CI. While CI was initially aimed at adult populations, there has been a notable shift towards pediatric applications. This transition is not only indicative of the technological reliability of CIs but also underscores their potential in drastically improving the life of younger individuals who might otherwise struggle with lifelong hearing impairments.

However, one important gap that our study revealed is the relative lack of focused research on the aging population. Given the global demographic trends of an increasing elderly population, this is a significant oversight. The elderly stand to gain not only from improved hearing but also from potential cognitive benefits. Numerous studies have shown a link between untreated hearing loss and cognitive decline, including an increased risk for dementia [40,41,42,43]. CIs could play a vital role in mitigating this risk by improving auditory perception, thereby enhancing social interaction and mental stimulation, which are key factors in cognitive wellbeing.

The safety and efficacy of anesthesia in the elderly population is an area that warrants further investigation. While general anesthesia is commonly used in CI surgeries, its risks increase with age, including potential complications such as post-operative cognitive dysfunction (POCD) [44,45,46]. Local anesthesia presents an alternative that could minimize these risks. It offers the advantages of quicker recovery times and reduced systemic effects, making it potentially more suitable for older patients. However, local anesthesia is not without its challenges, such as the need for more extensive patient cooperation during the surgery, and there is a large body of research specifically examining its suitability and effectiveness in CI procedures for the elderly.

Our study also highlighted the increasing volume of research around various etiologies for SNHL like genetic factors, infections, and tinnitus [4,47,48,49,50,51,52]. This suggests a broadening scope of CI application, making it increasingly relevant for a wider range of SNHL causes. In particular is the increase in publications around single-sided deafness and asymmetric hearing loss, which have previously been less traditional candidates for CI [53,54,55,56,57,58,59,60,61].

In addition, we have identified shifts in the number of publications focusing on bilateral and asymmetric hearing loss. Beginning in 2014, there was a marked increase in articles centered on asymmetric hearing loss. This trend suggests a growing scientific and clinical interest in asymmetric hearing loss as a new indication for cochlear implantation which has led to a shift from bilateral to asymmetric loss. This can be reflected in the study by Van de Heyning et al. (2015) [62], which emphasized the importance of cochlear implants for asymmetric hearing loss and presented a unified testing framework for single-sided deafness. The complexities associated with CI in patients with asymmetric hearing loss—such as the long-term absence of ear stimulation and the competition between cochlear implants and acoustic hearing in the other ear —have likely warranted targeted research.

From a technological standpoint, the emphasis on hearing preservation is prominent, aligning with the medical principle of ‘first, do no harm’ [63,64,65,66,67,68,69,70,71]. Furthermore, the advent of robotic guidance techniques in CI surgeries point to an exciting future where precision and automation could redefine surgical outcomes [72,73]. Recent trends also show a growing interest in alternative methodologies, such as the use of dexamethasone-eluting electrode arrays and local anesthesia, which may offer additional benefits, especially for older patients [74,75,76].

Another observation from our study is the interdisciplinary and growing nature of CI research. Not only did we note a surge in the overall volume of CI publications over the last five decades, but we also saw an increasing trend of these publications appearing in journals outside the scope of Otolaryngology. This suggests a broader scientific and clinical interest in CI technology, affirming its status as a transformative bio-technological solution for sensory organ deprivation. The escalation in publication volume indicates an expanding field that is drawing attention from diverse sectors of the scientific community. The increased presence of CI research in non-ENT journals underscores the technology’s far-reaching implications beyond the realm of auditory sciences, affecting fields like neuroscience, geriatrics, and even psychology. This multidisciplinary interest not only enriches the knowledge surrounding CIs but also opens up access for cross-specialty collaborations that could further advance the technology.

Our study has several limitations, primarily related to the depth of content analysis and potential bias introduced by the search terms. The study’s scope is inherently constrained by these pre-defined topics and search terms, which do not cover all possible facets of CI research. Moreover, the inability to search within full-text articles might have resulted in some relevant publications being missed. Another limitation is that our study does not delve into the post-operative rehabilitation process, which is a critical factor in the overall success and efficacy of cochlear implantation. The rehabilitation journey, involving auditory training and therapy, is vital for optimizing the benefits of CI and, therefore, deserves its own focus in future studies.

## 5. Conclusions

To conclude, our analysis provides a focused view of the evolving landscape of CI research. While the observed dominancy of pediatric CI research among other age groups is encouraging, the relative neglect of the aging population presents a gap that needs to be addressed. Emerging trends in CI methodology and the cross-disciplinary impact of CI research are promising indicators of the technology’s future potential. A substantial rate of CI publications was in non-otolaryngology journals, suggesting the impact of this surgical solution on additional medical and scientific fields.

## Figures and Tables

**Table 1:** The terms list used to classify entries for comparison.

Category	Terms
Patient’s age group	Elderly/geriatric/octogenarians/nonagenarians
	Adults
	Pediatric/young/newborn/congenital
Hearing-loss etiologies	Tinnitus/hyperacusis
	CMV/TORCH/intrauterine infection/meningitis/labyrinthitis
	Syndromic hearing loss/non-syndromic hearing loss/genetic hearing loss/connexin/Usher/Waardenburg/Pandered/Wolfram syndrome/Stickler syndrome/GBJ2/GBJ6
	Auditory neuropathy spectrum disorder/auditory neural asynchrony
Indications for CI	Single-side deafness
	Asymmetric hearing loss
	Bilateral severe hearing-loss
Surgical methods	Hearing preservation
	Robotic/Robot-Assisted/Robotics
	Drug-eluting electrode/eluting electrode
	Electro-acoustic stimulation/electroacoustic stimulation
	Local anesthesia

CMV: Cytomegalovirus; TORCH: Toxoplasmosis, Other (syphilis, varicella-zoster, mumps, parvovirus, and HIV), Rubella, Cytomegalovirus, and Herpes simplex; GJB2 mutation: Gap Junction Beta-2 (also known as Connexin 26); GJB6 mutation: Gap Junction Beta-6 (also known as Connexin 30)

**Table 2:** Publications trends in Indications for CI and Hearing-Loss Etiologies.

Years	Hearing-Loss Etiologies			Indications for CI			
	Asymmetric Hearing-Loss	Bilateral Severe Hearing-Loss	Single Side Deafness	Tinnitus	Infection	Genetic Hearing-Loss	Auditory Neuropathy Spectrum Disorder
1972–1975	0	0	0	0	1	0	0
1976–1979	0	0	0	1	1	0	0
1980–1983	0	0	0	3	4	0	0
1984–1987	0	0	0	9	10	1	0
1988–1991	0	1	0	12	19	0	0
1992–1995	0	2	0	19	33	13	1
1996–1999	0	2	0	9	34	11	0
2000–2003	0	6	0	22	42	21	0
2004–2007	1	8	0	26	72	50	0
2008–2011	1	10	0	45	101	60	10
2012–2015	15	11	0	90	103	73	39
2015–2018	32	20	5	122	92	78	35
2019–2021	25	11	0	116	73	71	29

## Data Availability

All datasets generated during the current study are available from the corresponding author on reasonable request.
